# Intimate partner sexual aggression against Chinese women: a mixed methods study

**DOI:** 10.1186/1472-6874-14-70

**Published:** 2014-05-25

**Authors:** Agnes Tiwari, Denise Shuk Ting Cheung, Ko Ling Chan, Daniel Yee Tak Fong, Elsie Chau Wai Yan, Gloria Ling Lee Lam, Debbie Hoi Ming Tang

**Affiliations:** 1 School of Nursing, Li Ka Shing Faculty of Medicine, The University of Hong Kong, 4/F, William M.W. Mong Block, 21 Sassoon Road, Pokfulam, Hong Kong, China; 2 Department of Social Work and Social Administration, The Centennial Campus, The University of Hong Kong, Room 534, Jockey Club Tower, Hong Kong, China; 3 Po Leung Kuk, Hong Kong, 66 Leighton Road, Hong Kong, China

**Keywords:** Intimate partner, Sexual aggression, Mental health, Women, Chinese

## Abstract

**Background:**

Although intimate partner sexual aggression has been shown to be associated with adverse mental health outcomes, there is scant information about sexual aggression in Chinese intimate relationships in general and about its mental health impact in particular. This article aimed to investigate sexual aggression in Chinese intimate relationships, including the use of force or threat of force and non-physical coercive tactics in unwanted sex.

**Methods:**

The quantitative and qualitative data used in this paper were drawn from a prospective cohort study conducted in Hong Kong between September 2010 and September 2012. A total of 745 Chinese women aged 18 or older who had been in an intimate relationship in the preceding 12 months were recruited from sites in all districts of Hong Kong. Multiple logistic regression analysis, ordinary linear regression, and *t*-tests were used in quantitative analysis. Directed content analysis was used to analyze the transcripts of 59 women who revealed experiences of intimate partner sexual aggression in individual in-depth interviews.

**Results:**

Of the 745 Chinese women in the study, 348 (46.7%) had experienced intimate partner physical violence in the past year, and 179 (24%) had experienced intimate partner physical violence and sexual aggression in the past year. Intimate partner sexual aggression significantly predicted PTSD and depressive symptoms after controlling for intimate partner physical violence. Among the 179 women reporting intimate partner physical violence and sexual coercion in the past year, 75 indicated that their partners used force or threat of force to make them have sex, and 104 of them reported that they gave in to sex because of non-physical coercive tactics used by their partners. Qualitative data revealed a variety of non-physical coercive tactics with different degrees of subtlety used to coerce women into unwanted sex with their partners. Chinese women experiencing physically forced sex had significantly more depressive symptoms and PTSD symptoms.

**Conclusions:**

Our findings indicate that sexual aggression in Chinese intimate relationships has specific mental health consequences over and above those associated with physical violence. Assessment of partner violence in Chinese relationships should include screening for sexual aggression in order to provide appropriate interventions.

**Trial registration:**

ClinicalTrials gov NCT01206192

## Background

Intimate partner sexual aggression is defined as the use of psychological, verbal, or physical coercion to obtain or attempt to obtain sexual contact with an intimate partner who is unwilling or unable to consent [1]. Intimate partner sexual aggression has received increasing attention over the past two decades. Specifically, an evolving body of research literature has shown that sexual aggression is not only a marker of severe physical violence in intimate relationships, it is also problematic in its own right and is associated with specific consequences [2]. For example, compared with intimate partner physical violence, intimate partner sexual aggression has been found to be associated with a higher risk of homicide and more deleterious health outcomes [3]. Intimate partner sexual aggression has also been identified as a significant predictor of post-traumatic stress disorder (PTSD), even after controlling for the severity of physical violence [4,5].

Sexual aggression in intimate relationships is not a single phenomenon. Rather, a spectrum of sexually aggressive acts has been documented [2]. Some are unwanted sexual acts obtained through the use or threat of force [2]. Such physically forced sexual acts are considered by some to qualify as rape [6-8] and can be identified not only by the use or threat of force by the perpetrator, but also the lack of consent by the victim [9]. Identifying acts of unwanted sex in intimate relationships where there is no report of forced sex or lack of consent, however, is more problematic. The term “sexual coercion” has been used to describe a woman’s acquiescence to her partner’s demands for sex [8,9], and the partner may not even know that the sex is unwanted [9]. As such, non-physical sexual coercion is often subtle and requires careful assessment of how a woman gives in to sex without the partner using force or threat of force [9]. It has been suggested that quantitative measures may not adequately reveal the context surrounding intimate partner sexual aggression in general [10] and especially the subtle forms of non-physical sexual coercion [9]. Supplementing quantitative self-report measures with in-depth interviews will more likely reveal the nuances of the complex phenomenon of intimate partner sexual aggression, particularly for identifying victims who are at greater risk of sexual aggression-specific outcomes [2].

Eliciting information from women about their experiences of intimate partner sexual aggression, irrespective of whether force has been used, is further complicated by cultural factors. Specifically, in cultures such as the Chinese culture, where sex is a taboo subject [11] and wives are socialized to believe that they should satisfy their husbands sexually [12], extricating experiences of unwanted sex from the women requires special skills. Previously, we adopted the method of empathetic interviewing to help Chinese women recount their experiences of intimate partner violence, including sexual aggression [13]. Among the narratives provided by the women reporting intimate partner physical violence victimization were accounts of how their partners also used various coercive acts, including the use of force, to make them have sex. The findings indicated that it was possible to elicit personal experiences of intimate partner sexual aggression from Chinese women, and that there was a need to further investigate the relative effects of physical violence and sexual aggression on these women’s health in light of the findings in the Western literature [4,14].

In addition, although the use of force or threat of force by an intimate partner to gain sexual access has long been recognized as a vicious and extremely damaging form of sexual aggression [8,15], less is known about the more subtle forms of unwanted sex where no force or threat of force is used [9]. An exception is a study by Basile [9], in which five types of acquiescence to unwanted sex in marital contexts were identified in a group of women recruited from a national telephone poll in the United States. The study uncovered the processes, including subtler processes, through which women gave in to unwanted sex in intimate relationships. Basile’s study also highlighted the need to recognize the subtler forms of sexual aggression, suggesting that these may be precursors to more severe forms. To date, information on the subtler forms of intimate partner sexual aggression is still limited, and even less is known about the differential impact on health made by physically forced sex and unwanted sex obtained by non-physical coercion. Such information is important for identifying those at risk so that tailored interventions may be provided for secondary and tertiary prevention.

For the purpose of this paper, we distinguish intimate partner sexual aggression based on whether force or threat of force was used in unwanted sex. Physically forced sex is defined as any unwanted sexual act (vaginal, anal, or oral penetration) that is obtained without consent through the use of force or threat of force [2]. Non-physical coerced sex is defined as any unwanted sexual act (vaginal, anal, or oral penetration) with a partner that a woman gives in to as a result of being “coerced, tricked, pressured, and bullied into having sex” [8], p.86. Non-physical coerced sex differs from physically forced sex in that no physical force or threat of force is used by the partner, and it does not involve the lack of consent and/or struggle by the woman [9]. Although we distinguish between physically forced sex and non-physical coerced sex for the sake of clarity when making comparisons, we are not suggesting that they are two distinct phenomena. Rather, we acknowledge that they are different forms of sexual aggression existing on a continuum of severity, and that mildly coercive sexual acts can become more severe over time [9], hence the need for a better understanding of the subtler forms of sexual aggression.

This article reports on sexual aggression in Chinese intimate relationships. Specifically, predictive factors of sexual aggression were identified, and the relative effects of physical violence and sexual aggression on mental health were examined. After distinguishing unwanted sex experienced by Chinese women based on whether physical force or threat of force was used by their partners, we then described the non-physical coercive tactics used to make the women give in to unwanted sex. The differential impact on the women’s mental health caused by physically forced sex and non-physical sexual coercion was also compared. The findings are useful for generating much-needed information about the different forms of unwanted sex in Chinese intimate relationships and about their impact on the women’s mental health. This information can help professionals to better identify the treatment needs of these often silent and under-served women and determine the most appropriate interventions to reduce further risks and promote recovery.

## Methods

### Study design

Data for this analysis were drawn from a prospective cohort study conducted between September 2010 and September 2012 that aimed to classify Chinese women’s experiences of intimate partner violence victimization based on Johnson’s Typology of Domestic Violence [16]. This paper reports specifically on the women’s experiences of intimate partner sexual aggression and the impact of these experiences on their mental health, assessed both quantitatively using a series of measurement tools and qualitatively through in-depth interviews. The study was approved by the Institutional Review Board of the of the University of Hong Kong/Hospital Authority Hong Kong West Cluster (HKU/HA HKW IRB: UW 10–095) on 1 March 2010.

### Participants and settings

A total of 745 women, including 527 who had been abused and 218 who had never been abused, were recruited from sites in all 18 districts of Hong Kong. Recruitment sites included community centers operated by non-governmental organizations, shelters for abused women, and Family and Child Protective Services Units under the Social Welfare Department of the Government of Hong Kong Special Administrative Region. Chinese women aged 18 and older who had been in an intimate relationship in the preceding 12 months were eligible to participate. Women who were unable to communicate in Cantonese or Putonghua (the two main dialects spoken in Hong Kong) or were not competent to give informed consent were excluded from the study.

For the quantitative part of this paper, the data of all 745 women were analyzed. Based on the findings of a previous local study [17] with an effect size of 0.14 in terms of differences in mental health outcomes among women experiencing physical violence, those experiencing sexual aggression in addition to physical violence, and those with no experience of partner violence, a minimum of 495 women were required for this study. This was calculated using a 5% level of significance with a power of 80% and was estimated using the software G* Power 3.1.7 (Franz Faul, University of Kiel, Kiel, Germany). Thus, the sample size of 745 women in the quantitative component of this paper should be adequate to detect the differences in mental health outcomes among never abused women, physically abused women, and physically abused women who have experienced sexual aggression victimization.

For the qualitative part of this article, we extracted personal experiences of intimate partner sexual aggression reported by abused Chinese women in individual in-depth interviews. In the prospective cohort study [16] from where the data for this paper were drawn, the first 200 participants were also interviewed individually for the purpose of instrument validation [18]. From the transcripts of these 200 women, we selected all of the interviews (n = 59) in which the women revealed that they had experienced unwanted sex in their intimate relationships.

### Data collection

#### *Quantitative data collection*

After obtaining written informed consent, the following quantitative measuring instruments were administered by an experienced researcher to each of the participants in a private room to ensure the women’s safety and confidentiality.

The Chinese Version of the Abuse Assessment Screen (C-AAS) is a scale consisting of five dichotomous yes–no questions designed to determine the use of psychological abuse, physical violence, and sexual aggression by a former or current intimate partner within a defined period of time (i.e., lifetime and past 12 months), and whether the respondent was afraid of the perpetrator. The C-AAS has been validated as an instrument with satisfactory measurement accuracy and utility to detect intimate partner violence in the Chinese population [19]. If the woman answered affirmatively to having experienced intimate partner sexual aggression within the past 12 months, she was also asked to indicate whether her partner used physical force or threat of force to make her have sex with him.

The Chinese version of the Revised Conflict Tactics Scale (C-CTS2) [20] was used to assess women’s experiences of physical violence victimization in the preceding year based on eight items which measured *moderate physical violence* (e.g., pushing, shoving, and grabbing) and *severe physical violence* (e.g., licking, biting, beating up, choking, and attacking with a weapon). The C-CTS2 has been validated and shown to have satisfactory validity and reliability for the Chinese population [21].

The Chinese version of the Revised Controlling Behaviors Scale (C-CBS-R) [18] was used to measure the use of controlling behaviors by the woman (self-reports) and by the partner (derived from the woman’s report on her partner) based on 32 items with seven subscales (economic control, threatening control, intimidating control, emotional control, isolating control, using children, and minimizing). A cut-off score of 1.145 has been shown to distinguish high from low levels of controlling behaviors in Chinese intimate relationships [18].

The Chinese version of the 17-item Post-traumatic Stress Disorder Checklist Civilian Version (C-PCL-C) [22] was used to elicit self-reports from respondents on three symptom clusters of PTSD (DSM-IV-TR) [23], namely *re-experiencing*, *avoidance,* and *hyperarousal*, in the preceding month. The C-PCL-C validated and shown to have satisfactory sensitivity and reliability in identifying PTSD symptoms among the Chinese population, and optimal diagnostic efficiency was also demonstrated based on the mixed scoring criteria with a minimum symptom score of 4 for individual items and a total score of 50 as the cut-off [23].

The Chinese version of the 21-item Beck Depression Inventory version II (C-BDI-II) [24] was used to measure depressive symptoms in the preceding two weeks. The C-BDI-II has been validated and demonstrated to have satisfactory validity and reliability [25].

An investigator-developed questionnaire was used to collect socio-demographic information from the participants including age (self and partner), marital status, number of children, place of birth, number of years living in Hong Kong, education level, employment status, financial hardship, and financial support received.

#### *Qualitative data collection*

For the 200 women from whom both quantitative and qualitative data were collected, individual, face-to-face, in-depth interviews were conducted at the same time as the quantitative instruments were administered. For those who answered “yes” to the item on intimate partner sexual aggression in the C-AAS (n = 59), an opening question was posed:

Has your partner ever made you feel uncomfortable in your sexual relationship with him?

If the woman responded affirmatively to the opening question, she would be asked to elaborate on her experience, using an incident of her choice if necessary:

Tell me what he did that made you feel uncomfortable.

This was followed by more prompting questions as appropriate, for example:

How did you respond to his demand for sex?

What would happen if you didn’t agree to have sex with him?

To help her further elaborate on the circumstances under which she gave in to have sex with her partner, the following questions were used, depending on her response:

Have you ever had sex with him when you really did not want to because you thought:

– *it was your duty to have sex with your husband when he wants to have sex?*

– *he expected sex from you in return for the money he gave you for housekeeping?*

– *he would pester you for sex until you gave in?*

– *he would have sex with another woman if you did not have sex with him?*

– *he would leave you for another woman if you did not do so?*

– *he would do and/or say something to hurt you?*

– *he would turn really nasty if you did not agree to have sex?*

– *he would use physical force on you to make you have sex?*

For the circumstance(s) identified, participants were asked to provide more details including when, where, and what happened, as well as how they felt during and after the unwanted sex. For those who reported that their partners used physical force or threatened to use physical force to make them have sex, they were asked to describe the circumstances under which the forced sex occurred and their feelings during and after the episode.

Building on our previous experience of eliciting personal information from abused Chinese women, we used an empathetic interviewing technique [13] to help the women to open up and feel more at ease when they recounted their experiences of unwanted sex. Specifically, we acknowledged their reported experiences, for example by making the following statements.

I can hear what you were going through.

Unwanted sex in marriage happens but often it is not recognized.

It is not your fault that it happens.

You have a right to decide whether you want to have sex or not even in marriage.

We also validated the women’s feelings, for example:

I would imagine that (referring to the episode) must have been so frightening.

You did a good job to protect yourself (and/or the children).

As in our previous experience of interviewing Chinese women, we encouraged them to describe their experiences in narratives and use folk beliefs and Chinese idioms to help them expound on the event(s). Field notes documenting the women’s non-verbal responses (e.g., facial expression, gesture, tone of voice, and emotional state) were also kept.

Each of the interviews lasted about 40 to 50 minutes. All except two interviews were digitally recorded with the women’s permission. On the completion of the interview, an individual de-briefing was conducted to minimize distress or harm to the woman as a result of talking and/or thinking about her abuse experience. If she was found to be in need for professional help, a referral would be made to her case (social) worker with her permission. If she did not have a case worker, she would be advised of the need to seek professional help. Under such circumstance, the interviewer would report to the site investigator immediately for follow-up action.

### Data analysis

#### *Quantitative data analysis*

Statistical analysis was conducted using SPSS, Version 20.0 (IBM Corp., Armonk, NY, USA). Chi-square and F-tests were used to compare the demographic characteristics, social-economic status, and intimate relationship experiences among the three groups of women, namely never-abused, physically abused without sexual aggression, and physically abused with sexual aggression.

To determine any associated risk factors, partner violence (physical violence, or physical violence with sexual aggression in the past year) was modeled with multiple logistic regression analysis as a function of each demographic and socio-economic factor. Model adequacy was assessed by the Hosmer–Lemeshow test. To determine the relative predictive nature of physical violence and/or sexual aggression for depressive and PTSD symptoms, ordinary linear regression was used to assess the effect of the physical violence and/or sexual aggression on the C-BDI-II and C-PCL-C total scores after adjusting for socio-demographic variables. The differential mental health effects of physically forced sex and non-physical sexual coercion were examined using *t*-tests.

#### *Qualitative data analysis*

All recorded interviews were transcribed into Chinese, and the transcripts of the 59 interviews identified to contain women’s experiences of intimate partner sexual aggression were further analyzed. Qualitative content analysis was used to examine the women’s experiences. Specifically, a directed approach (i.e., directed content analysis) was adopted based on the understanding that, although prior theory existed about the phenomenon of intimate partner sexual aggression, information about this phenomenon in Chinese women was incomplete and would benefit from further description [26]. Using prior theory, we began by identifying key concepts or variables as initial coding categories [27] and then determined the operational definitions for these categories. For example, Finkelhor and Yllo’s conceptualization of sexual coercion [8] and Basile’s [9] acquiescence to sex served as an initial framework to identify Chinese women’s experiences of unwanted sex in intimate relationships. This was conducted by the researchers reading each transcript carefully and highlighting all text that, on first impression, appeared to describe unwanted sex in intimate relationships. Then, all highlighted passages were coded using the predetermined codes. A new code was given to any text that could not be categorized using the initial coding scheme. By identifying categories with exemplars and by offering descriptive evidence, it was possible to determine whether the identified categories supported or contradicted the existing theory about the phenomenon of intimate partner sexual aggression. Three of the researchers (AT, GL, and KL) independently analyzed the qualitative data using the process of directed content analysis described above before sharing their analysis for scrutiny during repeated rounds of critical discussion and debate. The final results were derived when consensus was reached.

## Results

### Socio-demographic characteristics

Of the 745 Chinese women participating in this study, 218 (29.3%) had never been abused, 348 (46.7%) reported intimate partner physical violence victimization in the past year, and 179 (24%) reported intimate partner physical violence with sexual aggression victimization in the past year. The socio-demographic characteristics of the three groups are shown in Table 1. The groups differed significantly on all the variables except number of children.

**Table 1:** Demographic characteristics of participants

	**Never-abused (*n*** **= 218)**	**Past-year physical violence (*n*** **= 348)**	**Past-year physical violence with sexual aggression (*n*** **= 179)**	**χ**^**2**^**/F**^**a**^	***p*-value**
Age (years), mean (SD)	46.5 (10.8)	40.4 (10.2)	39.4 (9.6)	31.47***	0.00
Younger than partner (>20 years)	0 (0.0)	13 (3.7)	19 (10.7)	27.83***	0.00
Married	205 (94.0)	215 (62.1)	99 (55.6)	87.60***	0.00
Number of children (>2)	35 (16.1)	64 (18.4)	25 (14.0)	1.73	0.42
Education (≤9 years)	119 (54.6)	230 (66.1)	119 (66.5)	8.95*	0.01
New immigrant status (residing in HK < 7 years)	25 (11.7)	128 (37.0)	85 (47.8)	64.12***	0.00
Woman in paid job	78 (36.1)	99 (28.5)	45 (25.1)	6.26*	0.04
Partner in paid job	172 (80.4)	245 (71.6)	115 (66.1)	10.40**	0.01
Financial hardship in the past year	19 (8.7)	220 (63.2)	136 (76.0)	221.24***	0.00
Financial assistance in the past year	18 (8.3)	133 (38.2)	84 (46.9)	81.54***	0.00
Recruited from shelter	0 (0.0)	116 (33.3)	91 (50.8)	15.19***	0.00

Values are presented as *n* (%) unless otherwise indicated.

^a^F-test for Age and Chi-square test for other variables.

* = *p* < .05, ** = *p* < .01, *** = *p* < .001.

### Risk factors

The adjusted effects of factors associated with intimate partner physical violence and/or sexual aggression in the past year are summarized in Table 2. The risk of being physically and sexually abused by an intimate partner was 39% higher when the partner used a high level of controlling behavior on the woman. The Hosmer-Lemeshow test revealed a small Chi-square value (5.132; *p* > 0.05), indicating goodness of fit of the model (Table 3).

**Table 2:** Adjusted analysis of factors associated with past-year physical violence and past-year physical violence with sexual aggression

**Variable**	**Adjusted OR**	**95% CI**
Age (years)	1.00	(0.98 - 1.02)
Age difference (partner-woman)	0.57	(0.25 - 1.31)
Married	0.58*	(0.35 - 0.96)
Education	1.11	(0.72 - 1.72)
New immigrant status	0.86	(0.54 - 1.35)
Number of children	1.34	(0.74 - 2.42)
Woman in paid job	0.83	(0.52 - 1.35)
Partner in paid job	0.85	(0.51 - 1.40)
Financial hardship in the past year	0.99	(0.60 - 1.64)
Financial assistance in past year	0.67	(0.42 - 1.06)
Recruited from shelter	0.62	(0.36 - 1.06)
C-PCL-C	1.03**	(1.01 - 1.04)
C-BDI-II	1.00	(0.96 - 1.02)
C-CBS-R (partner to woman)	1.39*	(1.04 - 1.85)

* = *p* < .05, ** = *p* < .01.

Adjusted by age, age difference between the couples, marital status, education level, new immigrant status, number of children, employment status of the couples, whether the women had encountered financial hardship, whether the women had received financial support in the previous year and whether the case was recruited from shelter.

**Table 3:** Results of Hosmer-Lemeshow test

**Hosmer-Lemeshow test**	**Chi-square = 5.132 ( *p *-value: 0.743)**
-2 Log likelihood	569.547
Cox & Snell R Square	0.142
Nagelkerke R Square	0.198

### Relative effects on mental health

Table 4 shows the impact of intimate partner physical violence and/or sexual aggression on women’s mental health. Compared with the never abused group, both the physical violence group and the physical violence with sexual aggression group had significantly higher C-BDI-II scores (*p* = 0.009 and *p* < 0.001, respectively) and C-PCL-C scores *(p* < 0.001 for both groups). Furthermore, experiencing both physical violence and sexual aggression had a greater impact on the C-BDI-II and C-PCL-C scores, compared with physical violence only.

**Table 4:** Impact of intimate partner physical violence and/or sexual aggression on women’s mental health

**Type of abuse**	**C-BDI-II (*n*** **= 745)**			**C-PCL-C (*n*** **= 745)**		
	Effect*	**SE**	***P***	Effect*	**SE**	***P***
Physical violence with sexual aggression	-6.675	1.664	<0.001	-11.687	2.015	<0.001
Physical violence	-3.024	1.160	0.009	-6.243	1.404	<0.001
No abuse	0	-	-	0	-	-

*Adjusted by age difference between the couples, education level, new immigrant status, number of children, employment status of the couples, whether the women had encountered financial hardship and whether the women had received financial support in the previous year.

To determine the relative predictive nature of physical violence and sexual aggression for depressive symptoms, regression models were estimated. These regression analyses showed that the full model (with both physical violence and sexual aggression variables) explained nearly 59.5% of the variance (R2 = .595), with physical violence uniquely accounting for approximately 54.5% of the variance (ΔR2 = .545) beyond that explained by sexual aggression severity (ΔR2 = .050).

Similar regression analyses were performed for the relative predictive nature of physical violence and sexual aggression for PTSD symptoms. The results showed that the full model (with both physical violence and sexual aggression variables) explained nearly 40.7% of the variance (R2 = .407), with physical violence uniquely accounting for approximately 32.9% of the variance (ΔR2 = .329) beyond that explained by sexual aggression severity (ΔR2 = .078).

### Physically forced sex and non-physical sexual coercion

Among the 179 women reporting intimate partner physical violence with sexual aggression victimization in the past year when responding to the C-AAS, 75 indicated that their partners used force or threat of force to make them have sex. In the in-depth interviews, the women elaborated on the force or threat of force used by their partners to make them have sex. A 34-year-old, emaciated university graduate who had experienced physical violence and sexual aggression throughout her 5-year marriage described her experience:

Whenever my husband wanted sex, I had to let him have it. Otherwise he would hurt me… just like that time when he bit my nipple so badly that I had to go to the hospital.

A 26-year-old woman described forced sex with her partner as rape:

It was rape… the way that he forced it on me, violently.

In addition to forced sex being used by their partners, many of the women were also subjected to degrading sexual acts, as recounted by a 32-year-old woman:

It’s the humiliation… that’s really bad… twice it happened last year (she did not want to elaborate). I did not see a doctor because I could not bring myself to tell the doctor what I went through.

Some of the women expressed that they lived in fear. In an interview, a 50-year-old woman spoke about her fear:

He was very strong and I could do nothing to protect myself. I was so scared… that one day he would kill me.

The 34-year-old graduate who sustained injury to her nipple by her partner also spoke with trepidation even though she was in a shelter at the time of the interview:

*He told me that he would punish me* ***severely*** *(raised her voice) if he ever found me. And he would keep doing that until I am dead. I am very scared of him and what he might do to me and my daughter.*

Responding to the C-AAS item on intimate partner sexual aggression, 104 women reported unwanted sex without force or threat of force being used by their partners. In the in-depth interviews, the women described a variety of non-physical coercive tactics used to make them give in to sex. One of the tactics was pestering, as reported by a 32-year-old mother of two children:

He would pester (for sex) and sulk and pester all day, even in front of the kids. It was easier to let him have it to stop the pestering, sulking, and embarrassment…

Another tactic was to make the woman feel that it was her duty as a wife to satisfy his sexual needs. A 45-year old woman said:

He said it was my duty as his wife to satisfy him whenever he wanted it (sex) and whatever he wanted to do.

Retaliation was also used by some of the partners. A 32-year old woman who came from China one year ago said:

I dared not say no (to his sex demand) because I knew if he didn’t get his way he would throw me out. With nowhere to live, I would have to go back to China and give up my daughter to him.

Similarly, many of the women were quite clear about the consequences of not giving in to sex. To them, the consequences were often far worse than enduring the undesired sex, as described by this 38-year-old woman who experienced sexual aggression in both her previous and her current marriage:

It is far better to go along with him. If I don’t agree, he would leave… like that time when he got really mad at me… because I did not satisfy him. He left home and lived with his mistress for nearly a month.

In light of the literature suggesting that mildly coercive sexual acts in intimate relationships may become more coercive with time, we compared the duration of the abusive relationships between physically forced sex and non-physical sexual coercion. We found no significant differences in duration of abusive relationships between the two groups of women (*p* = 0.292) (Table 5).

**Table 5:** Duration of abusive relationships between physically forced sex and non-physical sexual coercion

**Characteristics**	**Non-physical sexual coercion**		**Physically forced sex**		**t**	***P***^**a**^
	**(n = 104)**		**(n = 75)**			
	**Mean**	**S.D.**	**Mean**	**S.D.**		
Duration of abusive relationships	5.142	4.407	6.034	6.200	-1.058	0.292

^a^p-values obtained by *t*-test.

### Differential impact of physically forced sex and non-physical sexual coercion on women’s mental health

Compared with women reporting non-physical sexual coercion, those experiencing physically forced sex had significantly higher C-BDI-II (mean difference = 11.826; *p* < 0.001) and C-PCL-C (mean difference =17.771; *p* < 0.001) scores (Table 6). It is worth noting that, although the mental health consequences for women experiencing non-physical sexual coercion were less severe than those for women experiencing physically forced sex, the former group of women had C-BDI-II scores (mean = 26.654; SD = 17.382) in the range of moderate depression (i.e., 20–28) and C-PCL-C scores (mean = 51.442; SD = 22.300) above the cut-off score of 50 for PTSD symptoms.

**Table 6:** Differential effects on mental health between physically forced sex and non-physical sexual coercion

**Characteristics**	**Non-physical sexual coercion**		**Physically forced sex**		**t**	***P***^**a**^
	**(n = 104)**		**(n = 75)**			
	**Mean**	**S.D.**	**Mean**	**S.D.**		
C-BDI-II	26.654	17.382	38.480	13.774	-5.073	<0.001
C-PCL-C	51.442	22.300	69.213	16.493	-6.128	<0.001

^a^p-values obtained by *t*-test.

In the in-depth interviews, women experiencing physically forced sex were often tearful when describing how they felt about being forced and humiliated during unwanted sex, as shown by this 49-year-old woman with 5.5 years of abuse, whose partner forbade her to have her hair done by a male hairdresser and insisted on accompanying her everywhere she went:

I have no choice…in anything…not even the color of my hair… psychiatrists… psychologists… drugs… none of these have helped me. Now, I live one day at a time.

Although no force was used in the sexual context, women experiencing non-physical sexual coercion also expressed negative feelings, as noted by this 32-year-old executive in a 5-year sexually coercive dating relationship:

I worry a great deal about not meeting his (sexual) expectations… I have thought about killing myself.

Even when the relationship had ended, the effect of the partner’s sexually coercive acts remained for some of the women. A 49-year-old woman who experienced non-physical sexual coercion for 10 years said during an interview:

I have nothing left now that he’s gone. It’s better to be dead. It’s all my fault.

## Discussion

This article focused on sexual aggression in Chinese intimate relationships. To the best of our knowledge, this is the first article to chronicle the nuances of Chinese women’s experiences of intimate partner sexual aggression based on quantitative findings enriched by qualitative accounts. It is also the first to report on the differential mental health effects between physically forced sex and non-physical sexual coercion among Chinese women.

We found that, after controlling for intimate partner physical violence, intimate partner sexual aggression significantly predicted PTSD and depressive symptoms. These results are consistent with those of prior studies involving non-Chinese participants, in which even in the context of ongoing physical violence, the experience of sexual aggression directly resulted in more severe PTSD or depressive symptoms [3,4]. Furthermore, we extended the existing literature by revealing that physically forced sex and non-physical sexual coercion had differential effects on Chinese women’s mental health. Specifically, those experiencing physically forced sex reported worse PTSD and depressive symptoms than did those experiencing non-physical sexual coercion. Our findings have clinical implications in that they underscore the importance for health and social service professionals to screen for sexual aggression when intimate partner physical violence is reported. Also, careful assessment should be made to ascertain whether forced sex has been used by the partner. Such information will assist clinicians to predict more accurately the severity of women’s post-trauma symptoms and provide interventions that are tailored for their specific needs.

From the women’s accounts, we uncovered a variety of non-physical coercive tactics used by the partners to make them give in to unwanted sex. The tactics varied in form and subtlety. Some of the types of tactics are analogous to the forms of coercion identified in the classic work of Finkelhor and Yllo [8] on conceptualization of sexual coercion. For example, corresponding to Finkelhor and Yllo’s social type of coercion [8], some women in our study reported feeling obliged to have sex with their partners even though it was unwanted because they were socialized to believe (and then reinforced by their partner) that it was their duty. Similarly, consistent with Finkelhor and Yllo’s interpersonal type of coercion [8], some women in the present study may have given in to undesired sex as a result of the nonviolent threats made by their partners (e.g., withholding affection if sex was not given). Furthermore, the coercive tactics identified in our study also differed in subtlety. Some of the tactics were less subtle, as the women had learned from past experience that the consequence of not giving in to sex (e.g., retaliation by partners if sexual demands were not met) was far worse than succumbing to unwanted sex. Some of the tactics, however, such as compelling women to give in to unwanted sex out of obligation as a wife, appeared to be subtler. It has long been suggested that sexual coercion exists on a continuum [6,28,29] and that mildly coercive sexual acts can become more dangerous and severely coercive over time [9]. The findings in the present study indicating that women experienced different forms of sexually coercive tactics with different degrees of subtlety seem to align with the proposition of continuums of sexual coercion [6]. Longitudinal research should be conducted to verify the possibility of a continuum but also to validate whether less severe coercion can, in fact, become more severe over time.

Despite the taboo and shameful nature of intimate partner sexual aggression in Chinese society [30], the women in this study were prepared to share their personal experiences when interviewed in an empathetic manner. Moreover, their descriptions of how they negotiated and endured unwanted sex with their abusive partners revealed their resilience and resourcefulness, similar to their Western counterparts [31]. We should also take note of the finding that the risk of being physically and sexually abused was 39% higher when the partner used a high level of controlling behavior in the intimate relationship. This finding lends support to the call to facilitate risk assessment and treatment planning by conducting a clinical assessment of the perpetrator’s intention and motivation when intimate partner sexual aggression is found [2].

This study has several limitations. Sampling bias is one of the limitations. As the participants were recruited from organizations that provided services for abused women, they were essentially clinical samples. Thus, the high percentage of partner violence victimization reported would not be representative for the population of Chinese women in general. Furthermore, because self-reports were used to collect the information, recall errors may have led to important facts being unreported and social desirability may have introduced bias in favor of the prevailing social norms. Future research could consider using multiple data sources such as social reports, health records, and biophysiological measures to enhance the quality and accuracy of the data. The study design is another limitation. Because of the cross-sectional design of this study, even though an association was found between mental health problems and intimate partner sexual aggression, no causal inferences can be drawn. In future studies, a longitudinal design with data collected at different points over time would allow a better determination of the relationship between the presumed cause (i.e., sexual aggression) and the presumed effect (i.e., mental health problems).

## Conclusions

Our findings indicate that sexual aggression in Chinese intimate relationships had specific mental health consequences over and above those associated with physical violence. Furthermore, compared with unwanted sex gained through the use of non-physical coercive tactics, partners’ use of force or threat of force in unwanted sex had significantly worse impacts on the women’s mental health. Assessment of partner violence in Chinese relationships should therefore include screening for sexual aggression as well as whether force has been used in unwanted sex. Such information would help to better meet the needs of those at high risk of danger and health deterioration by providing tailored interventions for secondary and tertiary prevention. Given the new knowledge that different forms of coercive tactics with different degrees of subtlety exist in Chinese intimate partner sexual aggression, longitudinal research is required to study the temporal relationship between minor and severe coercion over time.

## Abbreviations

C-AAS: Chinese Abuse Assessment Screen; C-CTS2: Chinese Revised Conflict Tactics Scale; C-CBS-R: Chinese Revised Controlling Behaviors Scale; C-PCL-C: Chinese Post-traumatic Stress Disorder Checklist Civilian Version; C-BDI-II: Chinese Beck Depression Inventory version II; HKSAR: Hong Kong Special Administrative Region; HKSKH: Hong Kong Sheng Kung Hui; IPV: Intimate partner violence; PTSD: Post-traumatic stress disorder; SPSS: Statistical Product for Social Sciences.

## Competing interests

The authors declare that they have no competing interests.

## Authors’ contributions

Conceptualisation and design of the study: AT, EKLC, DYTF, ECWY, DHMT. Data collection and analysis: AT, GLLL. Preparing and reviewing the manuscript: AT, DC, GLLL, DYTF, EKLC, ECWY, DHMT. All authors read and approved the final manuscript.

## Pre-publication history

The pre-publication history for this paper can be accessed here:

http://www.biomedcentral.com/1472-6874/14/70/prepub
